# Bidirectional Kazakh Sign Language prosody-aware translation using computer vision and speech recognition techniques

**DOI:** 10.3389/frai.2026.1835419

**Published:** 2026-05-18

**Authors:** Mukhtar Zhassuzak, Zholdas Buribayev, Maria Aouani, Ainur Yerkos, Aigerim Yerimbetova

**Affiliations:** 1 Institute of Information and Computational Technologies CS MSHE RK, Almaty, Kazakhstan; 2 Department of Computer Science, Al-Farabi Kazakh National University, Almaty, Kazakhstan; 3 School of Engineering and Information Technology, META University, Almaty, Kazakhstan

**Keywords:** human-computer interaction, Liquid Neural Networks, prosody prediction, sign language translation, speech synthesis

## Abstract

**Introduction:**

This study presents a bidirectional communication system designed to enhance interaction between hearing-impaired and hearing individuals using gesture recognition.

**Methods:**

The proposed framework integrates multiple components, including Kazakh Sign Language (KSL) gesture detection, closed-set sentence classification, speech recognition, and speech generation. The system is trained and evaluated using a dataset consisting of images and video recordings of KSL gestures together with audio data for Kazakh speech synthesis.

**Results:**

Experimental evaluation demonstrates that the overall system achieves a sentence classification accuracy of 92% across 12 closed-set sentence classes, while individual model accuracy did not fall below 87%. Compared to other approaches, the proposed method achieved competitive results in terms of accuracy on the collected dataset; however, inference speed comparisons are module-specific and measured as latency in ms/sample.

**Discussion:**

The results confirm the feasibility of the proposed approach as a proof-of-concept for improving accessibility and communication through automated sign language understanding. The system demonstrates practical applicability in bridging the communication gap between signers and non-signers, thereby promoting greater accessibility.

## 1 Introduction

According to statistics of World Health Organization (WHO) (https://www.who.int/publications/i/item/9789240020481), in 2025 every fifth person had problems with their hearing. It is expected that by 2050 every third person will face some degree in hearing loss. Nowadays there is a lack of existing methods and data for underrepresented sign languages, including Kazakh Sign Language (KSL). Previous work on KSL has explored a range of approaches, including parallel corpus (Yerimbetova et al., 2025), neural networks ensemble methods (Amirgaliyev et al., 2024) and sign to speech based on long short-term memory and natural language processing techniques (Amangeldy et al., 2023). Sign language recognition has been extensively studied across other language groups, with approaches spanning convolutional and recurrent architectures such as hybrid Convolutional Neural Network - Bidirectional Long Short-Term Memory (CNN-BiLSTM) models (Spandana et al., 2023), as well as more recent transformer-based methods including deep vision transformers (Kothadiya et al., 2023) and joint recognition-translation frameworks (Camgoz et al., 2020).

Sign languages are characterized by complex hand movements that are difficult for computer vision systems to accurately recognize. We propose an innovative deep learning pipeline specifically designed for robust recognition of KSL gestures. This approach combines bounding boxes and keypoints detection using the YOLOv8-pose model, and time-aware sequence processing for sentence and prosody prediction using Liquid Neural Network (LNN). While You Only Look Once (YOLO) models and Long Short-Term Memory (LSTM) (Rivera-Acosta et al., 2021) are frequently used in real-time sign language systems due to their fast and efficient inference, LNN are not that popular in such tasks. Nevertheless, LNNs are often used in tasks for sequence processing, which gives a hint that it might be a powerful tool in similar sign language recognition solutions and prosody prediction methods (Fernandez et al., 2014). This was studied and showed efficiencies in such approach.

For speech synthesis, the system integrates a text-to-speech architecture based on FastSpeech2 with the ability to control intonation parameters. FastSpeech2 is a non-autoregressive transformer-based model capable of generating high-quality speech with low latency (Ren et al., 2020). It is widely used for prosody-aware tasks, including emotional speech. In the proposed framework, prosodic characteristics predicted by LNN, including duration, pitch, and energy, are used as control parameters for FastSpeech2. This allows for precise manipulation of speech characteristics and ensures that the synthesized audio accurately reflects the linguistic and emotional context of the original gesture sequence. Same model was trained and used for automatic speech recognition task.

The integration of gesture recognition, sentence-level linguistic modeling, and supervised speech synthesis forms a complete end-to-end multi-modal pipeline. This unified system not only translates KSL gestures into text, but also generates natural speech with appropriate prosody, significantly increasing accessibility and communication between the deaf and hearing communities.

## 2 Materials and methods

### 2.1 Dataset collection

The development of the proposed bidirectional communication system required the creation of several specialized datasets corresponding to the different components of the pipeline: sign language gesture detection, gesture sequence recognition, and speech generation. The datasets were collected and organized to support training of computer vision and speech synthesis models.

For the gesture detection module, an image dataset of KSL gestures including two hands (Akarsha et al., 2025) was collected. The dataset consists of images containing one or two hands performing different gestures. Each image was manually annotated with bounding boxes around the hands and corresponding keypoints representing hand landmarks. In total, 21 keypoints were used to describe the spatial structure of each hand. These annotations were prepared in a format compatible with the YOLOv8-pose framework, enabling simultaneous detection of hand locations and keypoint estimation. The total number of samples for this model is 47,766, split into 38,213 training, 4,776 validation, and 4,776 test samples. Images were recorded using an iPhone under medium indoor lighting conditions with four participants. Annotations (bounding boxes and keypoints) were prepared using MediaPipe as a labeling aid and converted to YOLOv8-pose format. Augmentation was applied to 10% of the dataset and included brightness adjustment, hue variation, and blur; no geometric frame-level transformations (rotation, flipping, etc.) were used for augmentation. Signer-independent splits were not enforced in this dataset.

To train the gesture sequence recognition model based on a LNN architecture, a video dataset of complete sign language sentences was collected. Each video contains a sequence of gestures forming a meaningful phrase or sentence in KSL. The recordings capture natural variations in gesture execution, speed, and hand positioning. A total of 6,000 video sequences were used for training the LNN model, split into 4,800 training, 600 validation, and 600 test samples. Recordings were made by 6 participants using an iPhone under medium indoor lighting. Keypoint sequences were automatically annotated using the trained YOLOv8-Pose model. Augmentation consisted of random perturbation of keypoint group locations to simulate spatial variation. Signer-independent splits were not enforced.

For the speech generation component, a dataset of paired audio recordings and corresponding text transcripts was pre-processed based on existing Kazakh dataset (Mussakhojayeva et al., 2022). Each sample consists of a spoken Kazakh sentence aligned with its textual representation. A total of 24,656 audio-text pairs were used, split into 19,725 training, 2,466 validation, and 2,465 test samples. All recordings were from a single speaker; speaker-independent splits were not enforced. Text transcripts were manually annotated and cleaned. No data augmentation was applied to the audio data.

### 2.2 Dataset pre-processing

Prior to model training, all collected datasets underwent pre-processing procedures tailored to the requirements of each subsystem.

The gesture detection dataset was augmented to increase the diversity of training samples and improve model robustness. Data augmentation techniques applied to the gesture detection dataset included brightness adjustment, hue variation, and blur transformations. These simulate real-world variation in lighting and image quality. Geometric transformations such as rotation and flipping were not applied, as they may alter keypoint topology and introduce label noise. For the sequence recognition module, augmentation consisted solely of random perturbation of keypoint group spatial locations.

For the LNN model, the recorded sentence-level videos were first segmented into individual frames. From each frame, the hand regions were detected and keypoint coordinates were extracted using the trained pose detection model. As a result, each video was converted into a sequence of frames containing bounding box coordinates and corresponding keypoint values. These sequences represent the temporal evolution of gestures and serve as the input features for the sequence recognition model. The audio dataset used for speech synthesis was processed to ensure consistent quality and alignment with textual transcripts. Audio recordings were normalized and converted into a uniform sampling format. Text transcripts were cleaned and standardized to remove inconsistencies in punctuation and formatting. The processed audio-text pairs were then used to train the FastSpeech2 model for speech generation.

### 2.3 Sign language translation models

The proposed system performs sign language translation through a multi-stage pipeline consisting of gesture detection, feature extraction, and temporal sequence modeling. The architecture combines a pose estimation model based on YOLOv8-Pose with a sequence learning model implemented using LNNs. This combination enables the system to recognize hand gestures and interpret them as meaningful sentences in KSL. Gesture detection and hand keypoint extraction are performed using the YOLOv8-Pose architecture. This model extends the YOLO object detection framework by incorporating pose estimation capabilities, enabling simultaneous prediction of object bounding boxes and keypoint locations.

In the proposed system, YOLOv8-Pose is used to detect both hands in an image and estimate their spatial structure through a set of predefined keypoints. Each detected hand is represented by a bounding box and a set of 21 keypoints corresponding to anatomical landmarks such as finger joints and the wrist. These keypoints provide a compact representation of hand posture, which is essential for recognizing sign language gestures. The model processes each frame of the input image or video stream and outputs the coordinates of the detected bounding boxes and keypoints. Compared to traditional pose estimation approaches, YOLOv8-Pose offers real-time performance and high detection accuracy, making it suitable for interactive communication systems (Sapkota et al., 2025). In the conducted experiments, the YOLOv8n-pose variant was used, representing the lightweight configuration optimized for efficiency. Input images were resized to 640 × 640 pixels and training was performed for 100 epochs with a batch size of 16. Optimization was performed using stochastic gradient descent (SGD) with momentum following the default Ultralytics configuration, and a cosine learning rate schedule was applied with an initial learning rate of 0.01. The default YOLOv8 augmentation pipeline was used, which includes mosaic augmentation, scaling, and color space transformations. During inference, a confidence threshold of 0.25 and a non-maximum suppression Intersection over Union (IoU) threshold of 0.70 were applied.

The extracted keypoint coordinates serve as feature vectors that describe the configuration of the hands in each frame and are passed to the temporal gesture recognition module.

**Figure 1:**
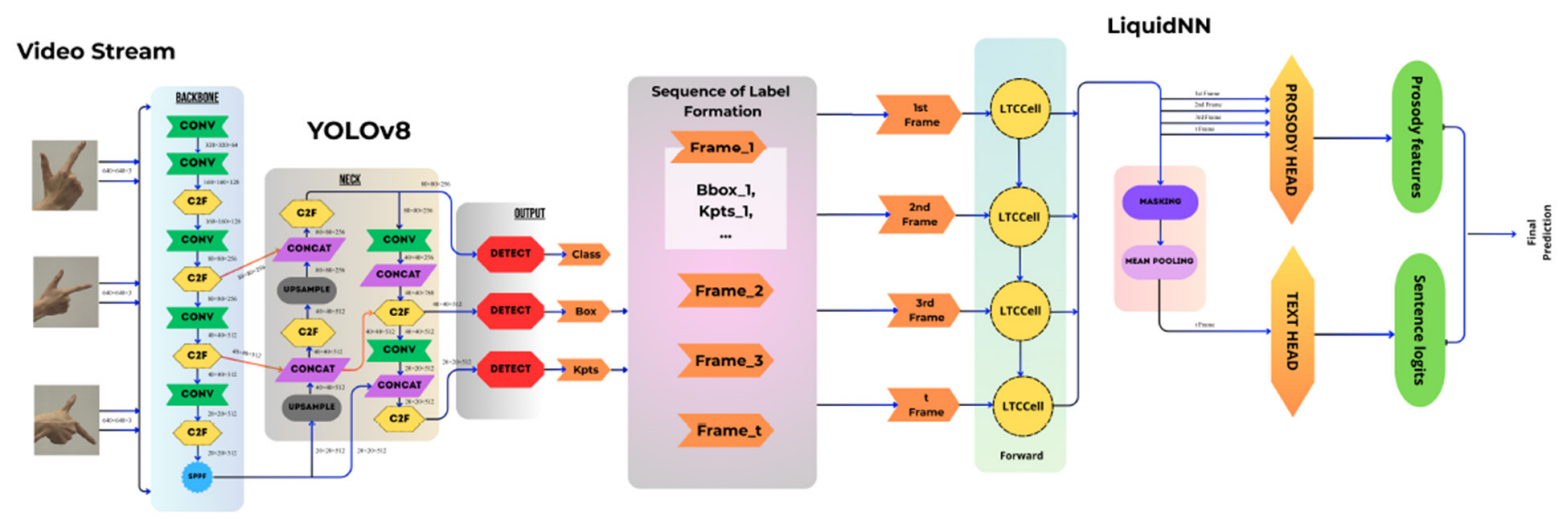
The architecture of the gesture (YOLOv8) and sentence (LNN) recognition models.

While individual frames provide information about static hand poses, sign language communication relies heavily on the temporal dynamics of gestures. To capture these dynamics, the system employs a LNN for sequence modeling (Figure 1).

LNNs belong to a class of neural architectures designed to model continuous-time dynamical systems. Unlike conventional recurrent neural networks, the internal dynamics of LNNs are governed by differential equations whose parameters can adapt over time (Hasani et al., 2021). This allows the network to learn complex temporal dependencies while maintaining stability and interpretability.

In the proposed framework, each video sequence representing a signed sentence is converted into a sequence of frame-level features consisting of bounding box coordinates and keypoint positions extracted by the YOLOv8-Pose model. These sequences are then fed into the LNN, which learns temporal patterns corresponding to different sign language phrases.

The LNN processes the sequence of gestures and outputs a predicted textual and prosodical representation of the signed sentence. Due to its ability to model long-term dependencies and adapt to variable-length inputs, the LNN is particularly suitable for interpreting continuous sign language communication. The LNN model consists of a single liquid layer with a hidden state dimension of 128. Neuron-wise learnable time constants were initialized to 0.5. Each input frame is represented by a 92-dimensional feature vector comprising 84 hand keypoint coordinates and 8 bounding box parameters. Sequences of up to 30 frames were processed with padding and masking to ensure that only valid frames contributed to the loss; temporal representations were aggregated using masked mean pooling. In addition to spatial features, auxiliary prosodic features (pitch, energy, and duration) were derived from keypoint dynamics and normalized per sequence. The model was trained under a multi-task learning framework jointly minimizing a cross-entropy loss for sentence classification and a masked L1 loss for prosody prediction, weighted by a factor of 0.3. Optimization used the Adam optimizer with an initial learning rate of 10^−3^; a ReduceLROnPlateau scheduler reduced the learning rate by a factor of 0.5 if validation accuracy did not improve for five consecutive epochs.

### 2.4 Speech models

To enable bidirectional communication between hearing-impaired and hearing individuals, the proposed system integrates speech technologies for both speech synthesis and speech recognition. The speech synthesis component converts translated text into natural-sounding speech, while the speech recognition component allows spoken input to be converted into text.

**Figure 2:**
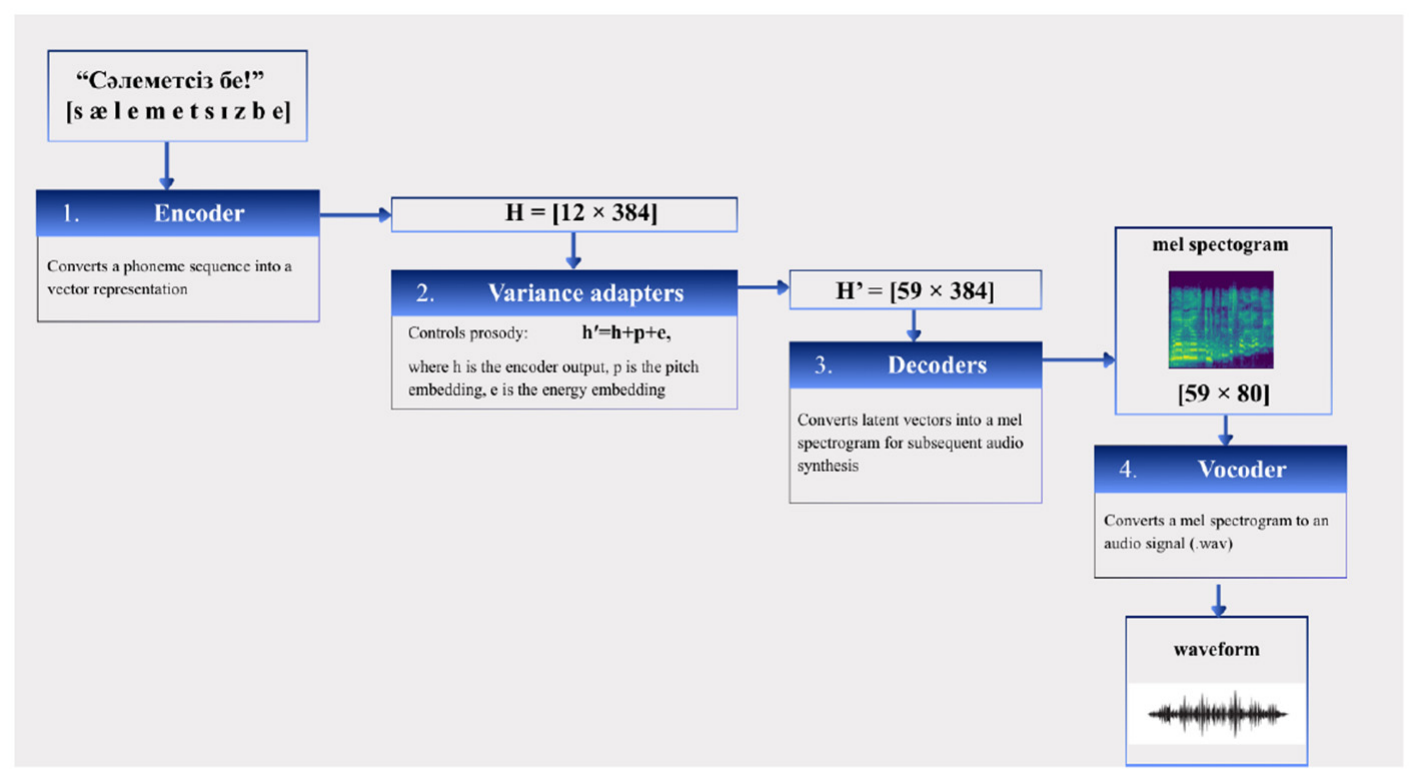
The working principle of the speech generation model (FastSpeech2).

Speech synthesis in the system is implemented using the FastSpeech2 architecture (Figure 2), a non-autoregressive neural network model designed for prosodical text-to-speech generation. The model employs a 4-layer Transformer encoder and decoder, each with a hidden size of 384 and 2 attention heads. A variance adaptor module explicitly models duration, pitch, and energy using convolutional predictors. Pitch was extracted using the Distributed Inline Object (DIO) algorithm and normalized via global mean-variance normalization; energy features were normalized in the same manner. The model generates 80-band mel-spectrograms, which are converted to waveforms using a High Fidelity Generative Adversarial Network (HiFi-GAN) vocoder. Training was performed using the Adam optimizer with a Noam learning rate scheduler and 4,000 warmup steps, implemented within the ESPnet framework.

FastSpeech2 addresses several limitations of earlier autoregressive Text-to-Speech (TTS) models by generating mel-spectrograms in parallel rather than sequentially. This significantly reduces inference time while maintaining high synthesis quality. The model incorporates variance predictors that explicitly model prosodic features such as pitch, duration, and energy, enabling more natural speech generation (Ikeda and Markov, 2024).

During training, the FastSpeech2 model learns the mapping between textual input and corresponding speech signals using paired audio-text data. The text output produced by the sign language translation module is converted into phoneme or character representations and processed by the FastSpeech2 encoder. The model then generates a mel-spectrogram, which is subsequently converted into a waveform using a neural vocoder.

By integrating FastSpeech2, the system can produce intelligible and natural-sounding Kazakh speech corresponding to the translated sign language sentences (Diatlova and Shutov, 2023). For automatic speech recognition (ASR), a Transformer-based encoder-decoder architecture was used, consisting of an 18-layer encoder and a 6-layer decoder with 8 attention heads and a hidden dimension of 512. Input features were 80-dimensional log Mel filterbanks augmented using SpecAugment, including time warping, frequency masking, and time masking. The model was trained using a hybrid CTC-attention loss with a CTC weight of 0.3, optimized with Adam and a warmup learning rate scheduler with 25,000 warmup steps. During inference, beam search with joint CTC-attention scoring was used for decoding.

**Figure 3:**
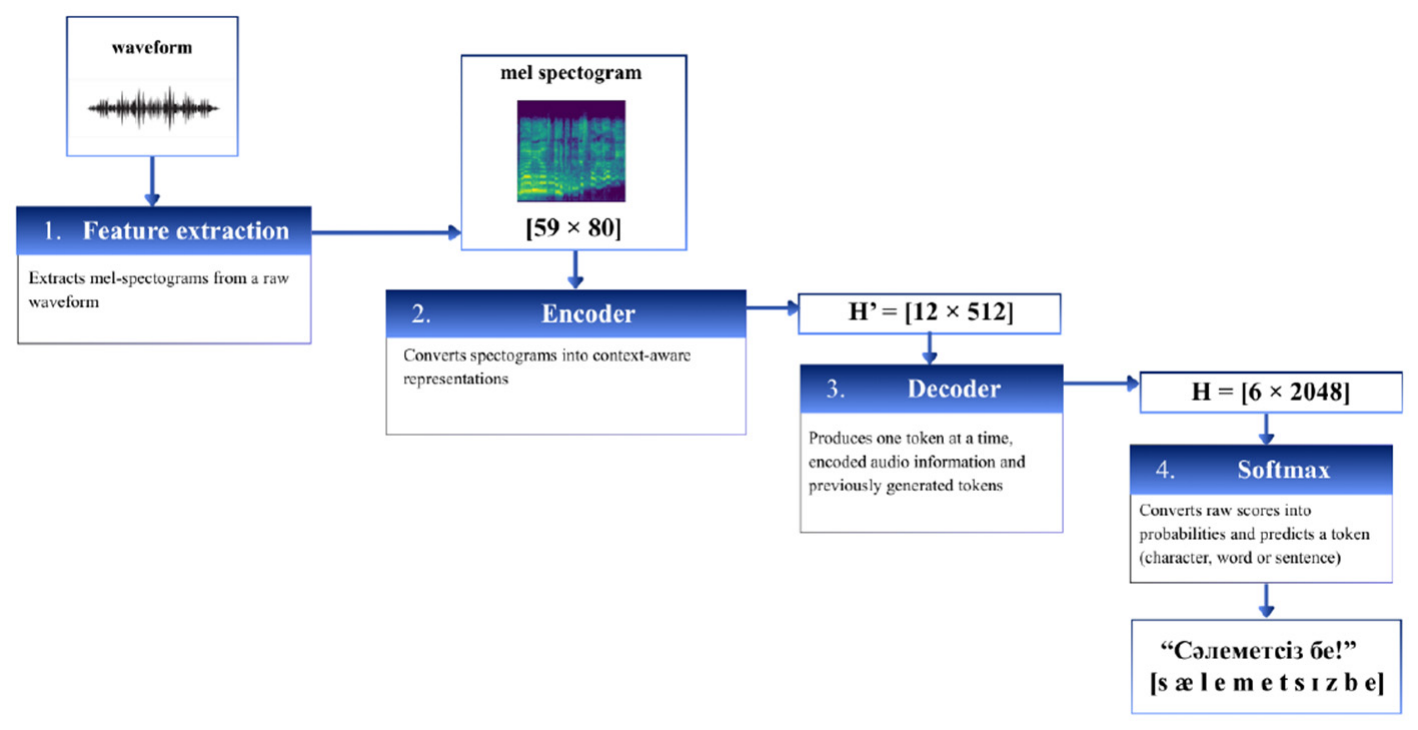
The working principle of ASR algorithm.

The ASR model processes audio input by extracting acoustic features from the speech signal and mapping them to corresponding textual representations (Figure 3). Speech signals are first transformed into spectral features that capture the temporal and frequency characteristics of the audio. These features are then processed by a neural recognition model that predicts the most probable sequence of text tokens.

The recognized text is then used as input for the sign language translation pipeline, allowing spoken messages to be conveyed to hearing-impaired users.

## 3 Results

The YOLOv8-pose model was trained on 100 epochs on a dataset of 17 classes presenting various Kazakh signs.

**Figure 4:**
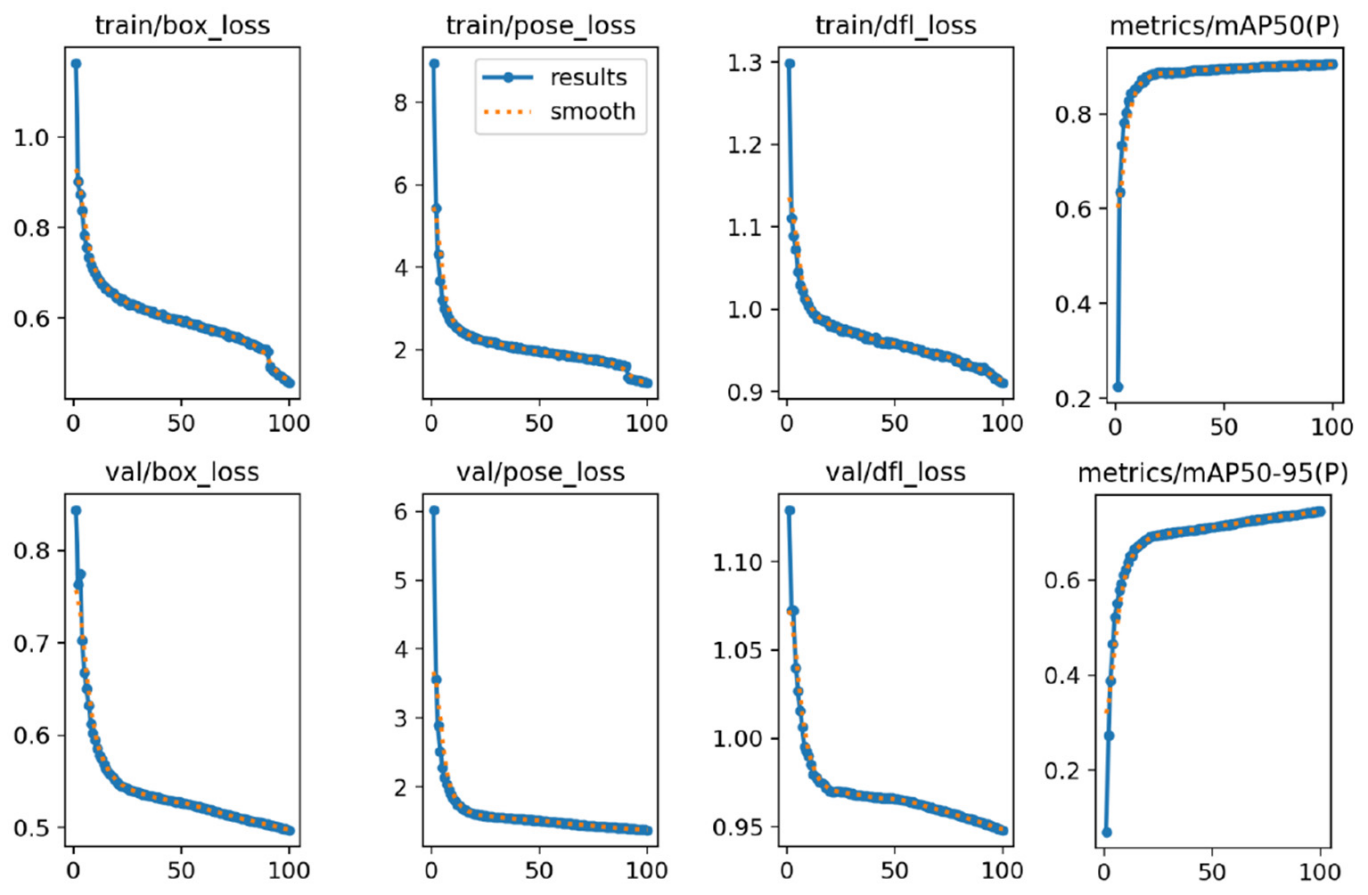
Training and validation results of YOLOv8-pose.

All six loss components across both training and validation splits demonstrate smooth and stable convergence with no signs of overfitting or instability (Figure 4). The training box loss dropped sharply within the first 20 epochs then continued to decrease gradually by epoch 100. The validation box loss followed a near-identical trajectory, converging from 0.85 to 0.49, confirming that the model's localization capability generalized well beyond the training set.

The pose loss was the highest-magnitude loss component. Both curves descended steeply in the early epochs and plateaued around 1.3–1.5 by epoch 100. The close alignment between training and validation pose loss throughout suggests the model learned robust keypoint regression without memorizing training samples. The DFL loss governs bounding box distribution refinement, converging smoothly to approximately 0.90–0.95 by the final epoch. The narrow gap between train and val curves reflects stable anchor-free detection learning.

The mean average precision at IoU threshold 0.50 rose sharply to approximately 0.87 by epoch 20, after which it continued increasing gradually, plateauing near 0.90 by last epoch. This rapid early-stage gain indicates that the model quickly learned to associate keypoint configurations with correct person detections. The averaged across IoU thresholds from 0.50 to 0.95 climbed to approximately 0.70–0.80 by epoch 100. While lower than the mAP@50 score, as expected given the stricter IoU criteria, the steady, monotonic rise without plateauing suggests the model was still improving in fine-grained localization precision at the end of training, indicating that additional epochs or learning rate scheduling could yield further gains.

**Figure 5:**
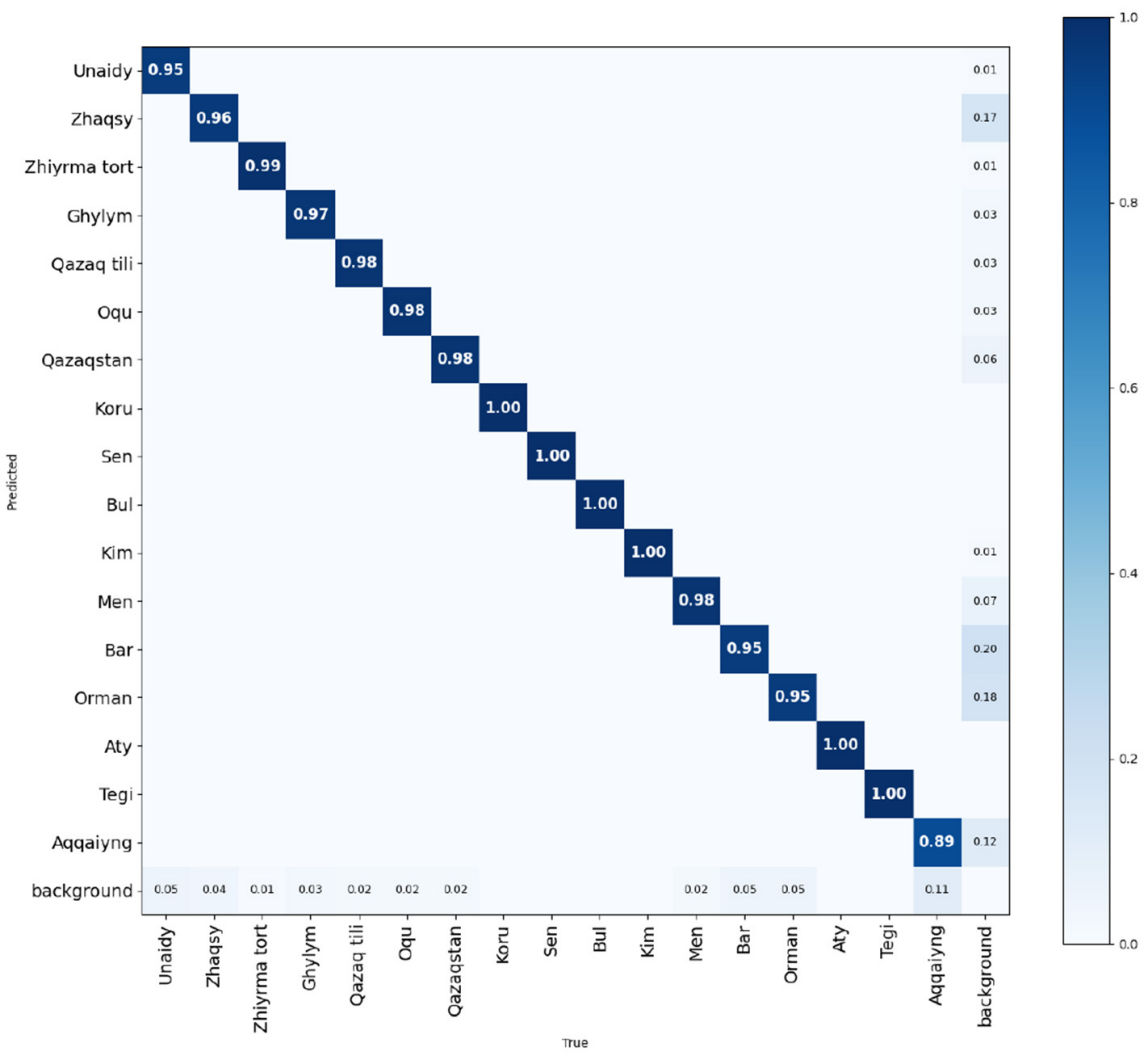
Normalized confusion matrix after YOLOv8-pose validation.

The normalized confusion matrix (Figure 5) reveals strong diagonal dominance across all classes, with the majority of gestures achieving recognition rates between 0.95 and 1.00, indicating that the YOLOv8-Pose keypoint detector reliably distinguishes most KSL words. The most notable source of confusion is the background class, which absorbs small fractions of several gesture classes, suggesting that low-motion or transitional frames are occasionally misclassified as no-gesture. The “Aqqaiyng” class shows the lowest true positive rate at 0.89, with 0.12 of its samples leaking into the background, the largest single off-diagonal error in the matrix. Conversely, five classes such as Koru, Sen, Bul, Kim, Aty, and Tegi, achieve a perfect score of 1.00 with zero misclassifications, demonstrating that gestures with sufficiently distinct keypoint configurations are learned without ambiguity. Overall, the confusion matrix confirms that the model generalizes robustly across the gesture vocabulary, with background boundary handling being the primary area for further improvement.

The LNN model was trained for 100 epochs on a dataset of 12 KSL sentence classes derived from skeletal keypoint sequences.

**Figure 6:**
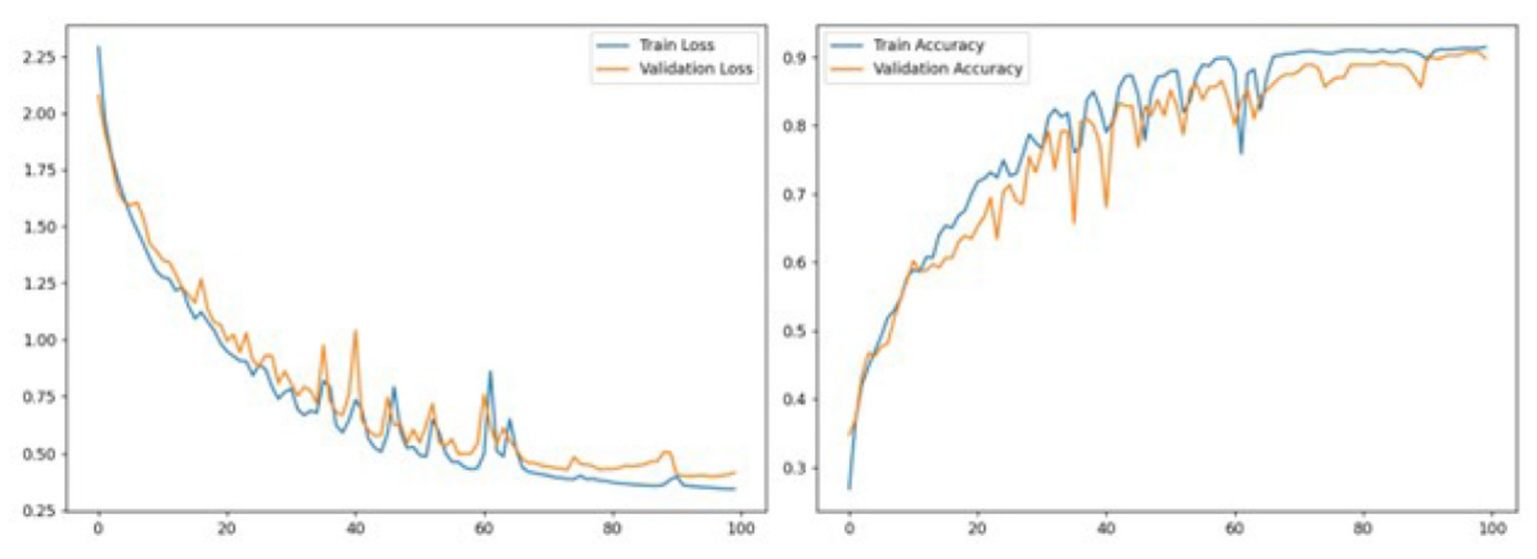
Training and validation loss and accuracy curves over 100 epochs for the LNN model.

Both training and validation loss (Figure 6) exhibited a consistent downward trajectory throughout training. The loss curves descended sharply during the first 20 epochs, indicating rapid feature acquisition in the early training phase. Beyond epoch 20, the curves continued to decrease more gradually, converging by epoch 100. The close alignment between the two curves throughout training suggests that the model did not suffer from significant overfitting, a notable strength attributed to the adaptive nature of liquid time-constant neurons.

Classification accuracy mirrored the loss dynamics in an inversely proportional manner. Training accuracy raised to approximately 0.91 at epoch 100, while validation accuracy reached 0.91 in parallel. The overlapping trajectories of training and validation accuracy throughout the learning curve confirm strong generalization of the learned temporal representations. Periodic oscillations observed between epochs 30 and 70 are consistent with the inherent stochasticity of the optimization process over challenging, variable-length gesture and prosody sequences, and these fluctuations dampen in the later epochs as the model stabilizes.

Macro-averaged precision, recall, and F1-score are 0.9421, 0.9029, and 0.9117, respectively; micro-averaged precision, recall, and F1-score are approximate to 0.9028 (Table 1). Each model was trained and evaluated over eight independent runs; best-run results are reported. Per-class results are presented in Table 2.

**Table 1:** YOLOv8-pose detection performance on validation and test sets.

Split	Precision	Recall	mAP@0.5	mAP@0.5:0.95
Validation	0.97	0.96	0.98	0.88
Test	0.96	0.95	0.97	0.86

**Table 2:** Per-class sentence classification results on the LNN test set.

Class	Precision	Recall	F1
Aqqaiyndy orman	1.00	0.75	0.86
Bul zhaksy	1.00	0.79	0.88
Bul kimdiki	0.94	0.88	0.91
Kim zhiyrma tort	0.50	1.00	0.67
Magan zhiyrma tort zhas	1.00	0.80	0.89
Magan Qazaq tili unaidy	0.92	0.92	0.92
Men gylymdy zhaksy koeremin	1.00	0.95	0.98
Men Qazaqstanda oqimyn	1.00	0.94	0.97
Sen gylymsyn	0.94	0.94	0.94
Senimen koeriseyik	1.00	0.94	0.97
Senin atyn	1.00	0.91	0.95
Qazaq tilin oqudy unataimyn	1.00	1.00	1.00

**Figure 7:**
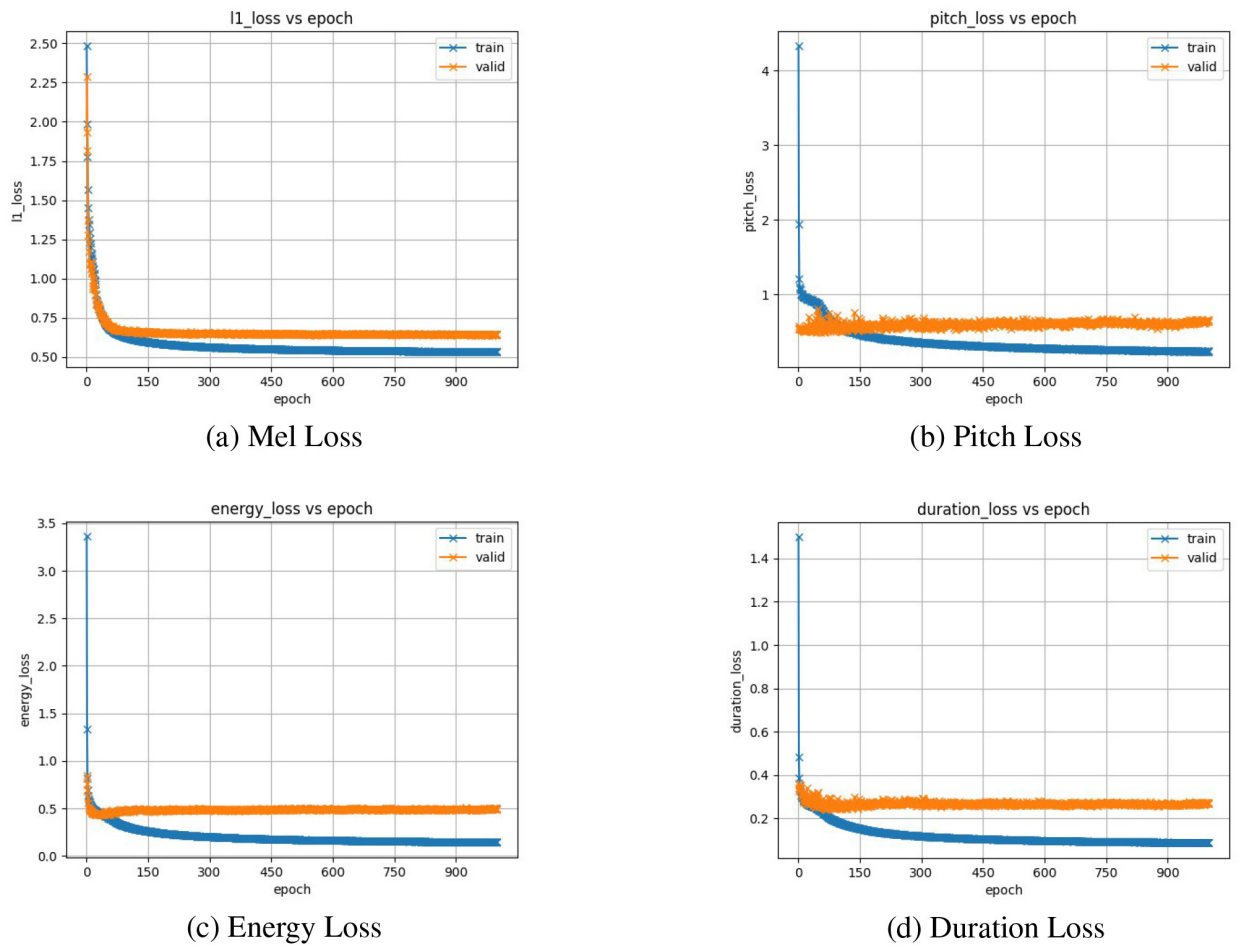
Training loss curves for mel-spectrogram, pitch, energy, and duration predictors in the FastSpeech2 model. **(a)** Mel Loss. **(b)** Pitch Loss. **(c)** Energy Loss. **(d)** Duration Loss.

The TTS system was evaluated using both subjective and objective metrics. An average Mean Opinion Score of 4.1 was obtained from eight participants over 20 audio samples. ASR-based intelligibility, specifically, Word Error Rate (WER) on synthesized outputs, was measured and achieved 24%. End-to-end TTS inference latency was 54 s total with a real-time factor of 10.8, measured on an NVIDIA RTX 3090, ASUS, China in FP16 with batch size 1, averaged over 1,000 utterances. Training and validation loss curves are presented and show stability and convergence (Figure 7). The ASR model achieves a WER of 15.61% and a Character Error Rate (CER) of 4.7% on the held-out test set. The relatively low CER is comparable to other systems reported for Kazakh (approximately 2–4%), while the higher WER reflects the agglutinative morphological complexity of the language.

The full sign-to-speech method was tested on 800 video samples and compared to other approaches, such as multilingual mT5 Transformers and LSTM (Table 3). The proposed method achieved the best results on the collected test dataset, reaching 92% accuracy. In terms of inference latency, mT5 achieves the lowest per-sample latency (10.2, ms/sample), while the proposed LNN model (19.2, ms/sample) offers a favorable balance between accuracy and speed. Although Transformers yielded competitive accuracy, the proposed LNN achieves considerable classification performance and remains suitable for real-time deployment. LSTM produced lower results, likely due to the variable-length nature of the video samples. While LSTM can be used with certain constraints such as setting predetermined video length, this limitation would also reduce the overall system speed. Accuracy is reported as the best result over 8 independent runs. Latency is measured as end-to-end inference time (ms/sample) on an NVIDIA RTX 3090 with FP16 precision, batch size 1, averaged over 1,000 samples after warm-up. The LNN latency covers keypoint feature extraction and sequence classification.

**Table 3:** Comparison of sentence classification methods.

Method	Accuracy	Latency (ms/sample)
LiquidNN (proposed)	92%	19.2
mT5	90%	10.2
LSTM	84%	20.8

## 4 Discussion

The proposed system demonstrates considerable effectiveness in automating the full pipeline of KSL recognition and speech synthesis, integrating YOLOv8-Pose-based keypoint extraction, LNN sentence classification, and FastSpeech2-driven text-to-speech generation with prosody control. The experimental results validate that each component of the pipeline contributes meaningfully to the overall system performance, with the LNN classifier achieving a macro F1-score of 0.91 across 12 sentence classes and the keypoint detector attaining mAP@50 of approximately 0.90, collectively establishing a strong foundation for real-world assistive communication applications. It is important to contextualize the scope of the proposed system. The gesture recognition module performs closed-set sentence classification over 12 pre-defined sentence classes, rather than open-vocabulary sign language translation. This distinction is significant: the system does not generalize to unseen sentences or morphological variants outside the training vocabulary. The use of the term “translation” throughout the manuscript refers to the broader pipeline goal of converting sign language gestures into spoken output; however, the underlying classification task is closed-set. Future work will need to extend the vocabulary and adopt open-vocabulary sequence to-sequence frameworks to achieve full translation capability.

A particularly noteworthy contribution of this work is the adoption of LNNs for temporal sequence classification including prosody features. Unlike conventional recurrent architectures such as LSTMs (Buribayev et al., 2025), SVM or GRU (Zholshiyeva et al., 2025), LNNs leverage continuous-time dynamics governed by ordinary differential equations, endowing the model with an inherent capacity to handle variable-length gesture sequences without explicit padding or segmentation. This architectural choice is reflected in the close alignment of training and validation metrics throughout the learning process, suggesting that the model captured the underlying temporal structure of sign gestures rather than overfitting to surface-level frame patterns.

The integration of FastSpeech2 for speech synthesis further extends the accessibility utility of the system by enabling not only intelligible speech output but also fine-grained prosody control over pitch, duration, and energy. This capability is especially significant for Kazakh, a low-resource language with limited publicly available speech synthesis tools, as it allows the generated speech to convey natural intonation rather than monotonic, robotic output. The combination of accurate gesture recognition with expressive speech generation thus brings the system closer to the communicative nuance required for practical deployment in assistive and educational contexts.

Nevertheless, several limitations warrant attention in future work. The most pressing concern is the computational latency of the speech generation module. FastSpeech2, while substantially faster than autoregressive models, still introduces non-trivial inference overhead when deployed on resource-constrained hardware, which may hinder real-time responsiveness in interactive settings. Optimizing the synthesis pipeline – through model quantization, knowledge distillation, or the adoption of lightweight vocoder alternatives (Guo and Zhang, 2024) – represents a critical direction for improving end-to-end system throughput.

Beyond inference speed, future efforts should address the current vocabulary constraints of the system. Word recognizers for American sign language can be scaled to sentence-level recognition with use of Natural Language Processing (NLP) techniques (Li et al., 2020). However, KSL needs a deep morphological study (Yin et al., 2023) and datasets in order to scale method to production. The present work is best understood as a proof-of-concept demonstration of a prosody-aware, bidirectional KSL pipeline. The gesture lexicon of 12 closed-set sentence classes is sufficient for this purpose but falls short of the breadth required for open-domain communication. Broader signer-independent and open-vocabulary evaluations would be necessary to move beyond proof-of concept status. Scaling the dataset to encompass a broader range of KSL expressions, including morphological variations and contextual sentence structures, will be essential for transitioning to a deployable assistive tool. Evidence from recent studies on Arabic (Abdul et al., 2021) and Indonesian (Caraka et al., 2025) sign languages further illustrates that recognition performance is highly language-dependent, underscoring the necessity of developing and evaluating dedicated approaches tailored to the linguistic and gestural characteristics of each sign language system.

Finally, the confusion matrix analysis highlighted that background frame misclassification remains a recurring source of error, particularly for gestures with low-motion characteristics. Incorporating temporal boundary detection or a dedicated silence/transition classifier as a pre-processing stage could mitigate this issue, improving both recognition precision and the fluency of the downstream speech output.

## 5 Conclusion

This study aims to improve accessibility and communication between hearing-impaired and hearing individuals by developing a bidirectional KSL recognition and speech synthesis system that exploits temporal keypoint dynamics and prosody-aware speech generation. In addition to system performance, this work sheds light on the individual contribution of each pipeline component, gesture detection, sequence classification, and speech synthesis, to the overall communication quality. For this purpose, we propose a multi-modal pipeline employing YOLOv8-Pose for hand keypoint extraction and a LNN for temporal sentence and prosody classification, which learns to interpret the spatial and temporal evolution of sign gestures as meaningful linguistic units.

While YOLOv8-Pose provides accurate frame-level spatial representations of hand configurations, the LNN captures the continuous-time dynamics of gesture sequences by modeling their temporal dependencies through differential equation-governed neurons, enabling robust sentence-level recognition without explicit sequence segmentation. The integration of FastSpeech2 with LNN-predicted prosody parameters further enables the system to produce natural, expressive Kazakh speech that reflects the linguistic and emotional context of the original gesture sequence. We evaluate our approach on a collected KSL dataset spanning 17 gesture classes and 12 sentence-level phrases, demonstrating that the proposed system achieves a gesture recognition mAP@50 of approximately 0.90 and a sentence classification macro F1-score of 0.91.

Furthermore, the bidirectional design with an ASR module for the hearing-to-deaf direction provides empirical evidence that the proposed YOLOv8-Pose and LNN-based framework can serve as a complete communication bridge without relying on large pre-existing Kazakh language resources or predefined gesture boundary annotations.

## Data Availability

The raw data supporting the conclusions of this article will be made available by the authors, without undue reservation.

## References

[B1] Abdul W. Alsulaiman M. Amin S. U. Faisal M. Muhammad G. Albogamy F. R. . (2021). Intelligent real-time arabic sign language classification using attention-based inception and bilstm. Comput. Electr. Eng. 95:107395. doi: 10.1016/j.compeleceng.2021.107395

[B2] Akarsha D. Hegde S. Yadav V. S. Kumar V. Srinivasa K. (2025). “Two hand gestures identification using deep learning techniques,” in 2025 5th International Conference on Emerging Research in Electronics, Computer Science and Technology (ICERECT) (Mandya: IEEE), 1–6. doi: 10.1109/ICERECT65215.2025.11378167

[B3] Amangeldy N. Ukenova A. Bekmanova G. Razakhova B. Milosz M. Kudubayeva S. (2023). Continuous sign language recognition and its translation into intonation-colored speech. Sensors 23:6383. doi: 10.3390/s23146383 37514679 PMC10385516

[B4] Amirgaliyev Y. Ataniyazova A. Buribayev Z. Zhassuzak M. Urmashev B. Cherikbayeva L. (2024). Application of neural networks ensemble method for the Kazakh Sign Language recognition. Bull. Electr. Eng. Inform. 13(, 3275–3287. doi: 10.11591/eei.v13i5.7803

[B5] Buribayev Z. Aouani M. Zhangabay Z. Yerkos A. Abdirazak Z. Zhassuzak M. (2025). Enhancing Kazakh Sign Language recognition with bilstm using yolo keypoints and optical flow. Appl. Sci. 15:5685. doi: 10.3390/app15105685

[B6] Camgoz N. C. Koller O. Hadfield S. Bowden R. (2020). “Sign language transformers: joint end-to-end sign language recognition and translation,” in Proceedings of the IEEE/CVF Conference on Computer Vision and Pattern Recognition (Seattle, WA: IEEE), 10023–10033. doi: 10.1109/CVPR42600.2020.01004

[B7] Caraka R. E. Supardi K. Kurniawan R. Kim Y. Gio P. U. Yuniarto B. . (2025). Empowering deaf communication: a novel lstm model for recognizing indonesian sign language. Univ. Access Inform. Soc. 24, 771–783. doi: 10.1007/s10209-024-01095-1

[B8] Diatlova D. Shutov V. (2023). Emospeech: guiding fastspeech2 towards emotional text to speech. arXiv preprint arXiv:2307.00024. doi: 10.21437/SSW.2023-17

[B9] Fernandez R. Rendel A. Ramabhadran B. Hoory R. (2014). “Prosody contour prediction with long short-term memory, bi-directional, deep recurrent neural networks,” in Interspeech (Singapore: ISCA), 2268–2272. doi: 10.21437/Interspeech.2014-445

[B10] Guo Z. Zhang J. (2024). “Fasttalker: jointly generating speech and conversational gestures from text,” in European Conference on Computer Vision (Milan: Springer), 177–194. doi: 10.1007/978-3-031-93806-1_14

[B11] Hasani R. Lechner M. Amini A. Rus D. Grosu R. (2021). “Liquid time-constant networks,” in Proceedings of the AAAI Conference on Artificial Intelligence, vol. 35 (New York, NY: Association for Computing Machinery), 7657–7666. doi: 10.1609/aaai.v35i9.16936

[B12] Ikeda M. Markov K. (2024). “Fastspeech2 based japanese emotional speech synthesis,” in 2024 IEEE 12th International Conference on Intelligent Systems (IS) (Varna: IEEE), 1–5. doi: 10.1109/IS61756.2024.10705252

[B13] Kothadiya D. R. Bhatt C. M. Saba T. Rehman A. Bahaj S. A. (2023). Signformer: deepvision transformer for sign language recognition. IEEE Access 11, 4730–4739. doi: 10.1109/ACCESS.2022.3231130

[B14] Li D. Rodriguez C. Yu X. Li H. (2020). “Word-level deep sign language recognition from video: a new large-scale dataset and methods comparison,” in Proceedings of the IEEE/CVF Winter Conference on Applications of Computer Vision (Seattle, WA: IEEE), 1459–1469. doi: 10.1109/WACV45572.2020.9093512

[B15] Mussakhojayeva S. Khassanov Y. Varol H. A. (2022). “KazakhTTS2: extending the open-source Kazakh TTS corpus with more data, speakers, and topics,” in Proceedings of the Thirteenth Language Resources and Evaluation Conference (Marseille: European Language Resources Association), 5404–5411.

[B16] Ren Y. Hu C. Tan X. Qin T. Zhao S. Zhao Z. . (2020). Fastspeech 2: fast and high-quality end-to-end text to speech. arXiv preprint arXiv:2006.04558. doi: 10.48550/arXiv.2006.04558

[B17] Rivera-Acosta M. Ruiz-Varela J. M. Ortega-Cisneros S. Rivera J. Parra-Michel R. Mejia-Alvarez P. (2021). Spelling correction real-time american sign language alphabet translation system based on yolo network and lstm. Electronics 10:1035. doi: 10.3390/electronics10091035

[B18] Sapkota R. Flores-Calero M. Qureshi R. Badgujar C. Nepal U. Poulose A. . (2025). Yolo advances to its genesis: a decadal and comprehensive review of the you only look once (yolo) series. Artif. Intell. Rev. 58:274. doi: 10.1007/s10462-025-11253-3

[B19] Spandana S. Madhura B. Sandhya A. Manish A. Kumar K. P. (2023). A hybrid cnn-bilstm model for continuous sign language recognition using iterative training. Int. J. Eng. Sci. Adv. Technol. 23.

[B20] Yerimbetova A. Sakenov B. Sambetbayeva M. Daiyrbayeva E. Berzhanova U. Othman M. (2025). Creating a parallel corpus for the Kazakh Sign Language and learning. Appl. Sci. 15:2808. doi: 10.3390/app15052808

[B21] Yin A. Zhong T. Tang L. Jin W. Jin T. Zhao Z. (2023). “Gloss attention for gloss-free sign language translation,” in Proceedings of the IEEE/CVF Conference on Computer Vision and Pattern Recognition (Vancouver, BC: IEEE), 2551–2562. doi: 10.1109/CVPR52729.2023.00251

[B22] Zholshiyeva L. Zhukabayeva T. Baumuratova D. Serek A. (2025). Design of qazsl sign language recognition system for physically impaired individuals. J. Robot. Control (JRC) 6, 191–201. doi: 10.18196/jrc.v6i1.23879

